# Comparison of the Functional Barrier Properties of Chitosan Acetate Films with Conventionally Applied Polymers

**DOI:** 10.3390/molecules25153491

**Published:** 2020-07-31

**Authors:** Andrea Walzl, Samir Kopacic, Wolfgang Bauer, Erich Leitner

**Affiliations:** 1 Institute of Analytical Chemistry and Food Chemistry, Graz University of Technology, Stremayrgasse 9/2, 8010 Graz, Austria; erich.leitner@tugraz.at; 2 Institute of Bioproducts and Paper Technology, Graz University of Technology, Inffeldgasse 23, 8010 Graz, Austria; samir.kopacic@tugraz.at (S.K.); wolfgang.bauer@tugraz.at (W.B.)

**Keywords:** migration testing, permeation, functional barrier, biopolymer, chitosan acetate, mineral oil hydrocarbons, gas chromatography, online-coupled HPLC-GC-FID

## Abstract

The current demand to cut back on the use of plastic materials has brought a major boost to the search for bio-based alternatives. Not only are plastic bags and primary food packaging under scrutiny here, but also those materials used as functional barriers to reduce, for example, the migration of mineral oil hydrocarbons (MOH) from recycled paper and board packaging. Most of the barriers now in use are synthetic, often have only moderate barrier functionalities and in addition reduce the environmentally-friendly character of cellulose-based materials. Against this background, bio-based polymers have been evaluated in terms of their functional barrier properties. Chitosan was found to be among the best performers in these materials. In this study, the behavior of a lab-made chitosan acetate film was compared with conventionally produced polymer films. The two-sided migration experiment described recently was used to determine the barrier properties of the tested materials. This not only allowed to test the intrinsic migration of the films and the permeation through them, but also to simulate real packaging situations by using a recycled paper as donor for MOH. The migrated fractions were determined using gas-chromatography-based techniques. While the conventionally produced polymer films showed only moderate barrier function, excellent results were seen for the biopolymer. It reduced the migration from the recycled paper to not detectable, singling it out as a good alternative to conventional materials.

## 1. Introduction

Europe is now making a concerted effort to cut back on the use of plastic-based food packaging materials [1,2]. Not only plastic bags and primary plastic-based packaging are targeted for replacement, but also many other plastics in use as e.g., bag-in-box materials or coating of paper and board food contact materials [3]. Bio-based polymers are one example of environmentally-friendly alternatives needed, showing good results in all kinds of applications [4].

Considering food contact materials (FCMs), paper and board are one of the most commonly used ones [5], due to their excellent functionalities: They offer the strength and stability needed for packaging, are of low weight and inexpensive. Even more important is the fact that cellulose-based packaging materials are renewable, biodegradable and recyclable and are, thus, considered to be excellent alternatives to plastic materials [3]. All cellulose-based FCMs have one major problem however, and this is that, due to their porous cellulose structure, they lack important barrier properties that are often required for long-term storage, such as resistance against moisture, oxygen, grease or volatile organic compounds. The latest need special attention in the context of recycled materials; recycled fibers are added, for example, in 90% of all produced folding boards, which are often used as secondary or tertiary packaging during transport and storage [6]. We face the problem that a huge pool of possible contaminants is created during the recycling process. These are not removed in this process, but are rather enriched in the final product, from where they can be transferred to the packed good during the period of use. Several authors have already discussed the migration and permeation of harmful compounds from and through such packaging into the food, concluding that the safety of the packed goods can no longer be guaranteed [7,8,9,10,11]. Among these contaminants are mineral oil hydrocarbons (MOH), which are divided into a saturated and an aromatic fraction. The mineral oil saturated hydrocarbons (MOSH) fraction contains branched and unbranched open-chain hydrocarbons (paraffins) and saturated cyclic hydrocarbons (naphthenes). The MOAH (mineral oil aromatic hydrocarbons) consist of highly alkylated aromatic ring compounds, which comprise up to 30% of the mineral oil fraction [12,13]. The health effects of these substances are still in discussion: While MOSH is accumulated in the human body, leading to organ weight increase, inflammatory diseases and micro granuloma formation, the MOAH may include mutagenic and carcinogenic substances [13,14,15].

Paper and board FCMs are, thus, unable to keep food safe over long storage periods and even add an additional source for contaminants in case of used recycled materials. So-called functional barriers have been discussed to be the only fast and practical solution for the mentioned problems [16]. They are placed between the packaging and the food, either by coating them directly onto the paper or by inserting them as an additional layer, thereby providing one main function: That of preventing any unwanted changes to the composition of the food [8]. This means that, on the one hand, they retain the quality, taste and aroma of foods, and on the other hand, they protect the food against contamination from the surroundings. Recently applied barriers include aluminum or different synthetic materials such as various polyolefins. Polyolefin films (e.g., polyethylene (PE) or polypropylene (PP)) are widely used in food contact applications and their barrier properties against migration from recycled board have been described by several authors. For example, the barrier efficiency of PP films with a thickness of 31–127 µm against migration from a recycled board into fatty and high-moisture food was evaluated in [17]. It was concluded that an increase of film thickness decreased migration of the surrogates, but still remained high. Testing was performed at 100 °C for 2 h using 10% ethanol, 95% ethanol and isopropanol as food-simulating solvents [17]. A further study investigated the permeation of surrogate substances through films using migration cells. PE and PP were mostly used in multilayer materials with a film thickness of up to 50 µm. It was concluded that high-density PE and oriented PP showed no barrier effect against permeation of organic compounds [16]. The same conclusion was derived in [18]; the taped barrier test was used to evaluate the barrier properties of internal bags included in paper and board packaging materials. They divided the barriers into five classes: Class 1 barriers showed no relevant effect and mainly included PE barriers, most of these being multilayers with a summed film thickness of about 50 µm. Class 2 included oriented PP barriers with a film thickness of 25–60 µm. The higher classes consisted of more complex multi-layer-multi-material barriers, also including PE and PP, while class 5 materials were complete barriers, such as aluminum foils [18]. A further study discussed the influence of the testing temperature. In contrast to the earlier studies, it was concluded that PP can be an efficient barrier for long-term storage at ambient temperature [19]. In conclusion, most studies showed that polyolefins lack satisfactory barrier properties. Therefore, they are often integrated into complex plastic-based multi-layer materials [18], resulting in additional waste, since recycling is often complex and expensive [3]. Their use thus goes against the trend to the change-over we currently face.

Bio-based alternatives are needed here based on both the increasing criticism of these complex plastic materials and the sustainability reputation of cellulose-based materials. One major disadvantage, which many of those materials have, is their water solubility. However, if only applied as a barrier against migration from secondary or tertiary (recycled) cellulose-based packaging and for dry non-fatty food, direct contact to moisture should not occur. Initial results of our studies have already been published and chitosan was identified to be one of the best performing materials [20,21,22]. It is a positively-charged polysaccharide, which is derived from deacetylation of chitin. Besides being edible and biodegradable, it is known to have excellent film-formation properties, forming robust and flexible films, due to the high crystallinity and strong hydrogen bonding between the molecular chains. The resulting films have good barrier properties against grease and oxygen [23,24]. In our earlier studies, it was shown that chitosan acetate has excellent barrier properties against the migration of MOH as well [21,22].

The present study has two main aims: First, the barrier properties of a free-standing lab-made chitosan acetate film are compared to industrially produced polyolefins, namely a 40 and 70 µm linear low-density PE film (LLDPE) and a 50 µm PP film. The method used to do so is the two-sided migration experiment using migration cells. The experimental set-up allows the testing of permeation through and migration from a sample into the food simulant poly 2.6-diphenyl-p-phenylene oxide (Tenax^®^ TA; MPPO) in a single experiment. Gas chromatography-based techniques are applied to characterize the migrated fractions: gas chromatography with flame ionization detection (GC-FID) is used to determine the overall migration of volatile compounds, gas chromatography with mass spectrometry (GC-MS) for quantification of permeation and online-coupling of high-performance liquid chromatography-gas chromatography with flame ionization detection (HPLC-GC-FID) is used to determine the migration of MOH. The experiment was only introduced shortly before and used for the screening of the barrier properties of coated paper samples [20,21,22]. Therefore, the second aim of this study is to extend the field of application to free-standing films and to further evaluate the methods capabilities.

## 2. Results and Discussion

As already mentioned, four barrier films were tested in a two-sided migration experiment to allow comparison of their barrier properties. Since the polymer films used are known to undergo changes when tested at higher temperatures, the experiments were performed using the standard conditions of 40 °C for 10 days [8]. Using this condition, no changes of the films were observed during the test period.

The two-sided migration experiment allowed the generating of complex information on the films by using the following procedure: The films were tested without an additional donor first, to obtain the migration of the initially present substances. For the polyolefin films, this were mainly polyolefin oligomeric saturated hydrocarbons (POSH). Second, a recycled fiber paper was used as donor and added to the experimental set-up, to obtain a real packaging situation: A recycled paper used as food packaging for dry, non-fatty food and a barrier film placed as an internal bag between the packaging and the food to prevent migration. The barrier properties of the films were then evaluated in terms of what and how much migrated into the food simulant MPPO from:

1. The packaging (= donor) without a barrier;
2. The barrier without a donor;
3. The packaging with a barrier.

The determined parameters were the overall migration of volatile substances using GC-FID, the migration of MOH using HPLC-GC-FID and the permeation of model substances through the tested samples using GC-MS. The results are presented below, starting with the overall migration. Table 1 gives the migration from the films without a donor (the intrinsic migration) and with applied donor. The determined values are normalized to the value of the donor.

**Figure 1:**
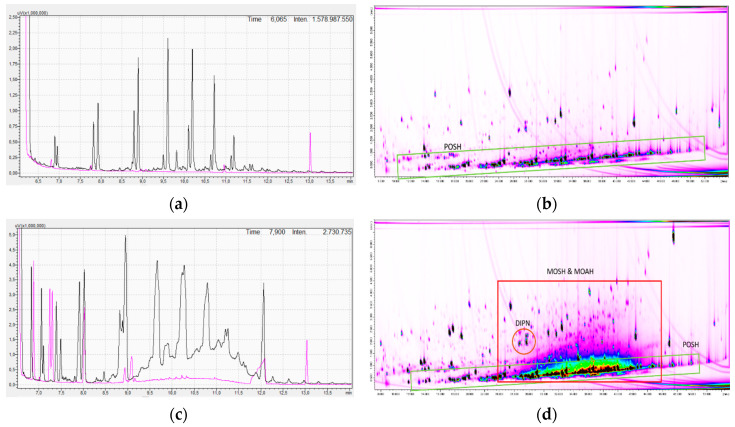
Comparison of high-performance liquid chromatography-gas chromatography with flame ionization detection (HPLC-GC-FID) and comprehensive 2D-GC×GC-MS (GC×GC-MS) chromatograms resulting from migration experiments (**a**) HPLC-GC-FID chromatogram of LLDPE film, (**b**) GC×GC-MS chromatogram of LLDPE film, (**c**) HPLC-GC-FID chromatogram of LLDPE film + donor, and (**d**) GC×GC-MS chromatogram of LLDPE film + donor.

Both LLDPE films showed a similar amount of migration than the donor. The combination of film and donor then showed a slight increase in the overall migration value. In contrast, the PP film had a 50% lower intrinsic migration than the donor and a 24% lower combined migration, indicating good barrier properties of the PP. The chitosan acetate film showed no detectable intrinsic migration and also reduced the migration from the recycled paper to not detectable. These results can be discussed in more detail by looking at the resulting chromatograms of, for example, the 40 µm LLDPE film, as given in Figure 1: Figure 1a gives the HPLC-GC-FID chromatograms of the film only. The black MOSH trace shows the typical pattern of POSH migrating from a PE sample (compare with [25,26,27]). The migration of these intrinsic substances is already in a similar concentration to that from the donor (not shown as single chromatogram). By contrast, no migrating substances can be seen in the MOAH trace (pink trace in Figure 1a). When performing the migration experiment with the recycled paper as donor the pattern changes, as given in Figure 1c: The POSH pattern of PE stays the same but is now overlaid by MOSH migrating from the recycled paper (black trace in Figure 1c). An unresolved hump is additionally formed, thereby overloading the chromatogram. The MOAH trace now also shows a hump, which has the same origin (pink trace in Figure 1c). The different patterns become even clearer when looking at the GC×GC-MS chromatograms in Figure 1b and d. Figure 1b gives the chromatogram of the intrinsic migration of the 40 µm LLDPE film. In contrast, Figure 1b shows the chromatogram of the migration of the same film, but with an additional donor from recycled paper and the resulting unresolved mixture of mineral oil.

**Figure 2:**
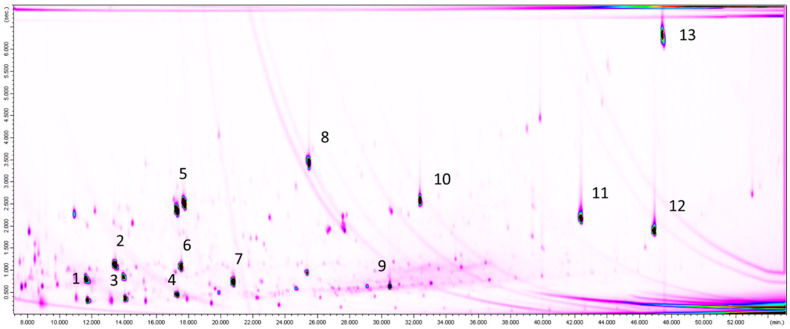
GC×GC-MS chromatogram of migration experiment using the chitosan acetate film and the recycled paper donor. Internal standards marked with numbers; (1) n-undecane (C_11_), (2) n-pentylbenzene (5B), (3) n-dodecane-d_26_ (d-C_12_), (4) n-tridecane (C_13_), (5) 1-and 2-methylnaphthalene (1-/2-MN), (6) cyclohexylcyclohexane (CyCy), (7) 1,3,5-tri-tert-butylbenzene (TBB), (8) benzophenone-d_10_ (d-Bzp), (9) n-nonadecane-d_40_ (d-C_19_), (10) di-n-butylphthalate-3,4,5,6-d_4_ (d-nBP), (11) bis-(2-ethylhexyl)phthalate-3,4,5,6-d_4_ (d-DEHP), (12) cholestane (Chol), (13) perylene (Per). Compare with Figure 2d.

In conclusion, the intrinsic migration from the polyolefin films stayed the same, but migration from the recycled paper was reduced, again indicating that the films may be a good barrier, although the overall migration value is high. For comparison, Figure 2 shows the resulting GC×GC-MS chromatogram of the chitosan acetate film, showing hardly any migrating substances except the added internal standards as marked.

To determine the barrier efficiency against migration of MOH from the donor, the HPLC-GC-FID data is used. When subtracting the intrinsic POSH migration of the films from the migration of donor + film, the precise level of migration through the barrier can be calculated. This was done for the barrier films and the results (giving the barrier efficiency) are presented in Table 2.

As can be seen in Table 2, the recycled paper shows a MOSH/MOAH ratio of about 90:10, which is a typical distribution [13]. Taking the frequently quoted draft of the mineral oil regulation into account, the migration of MOSH should stay <2 mg kg^−1^, the one for MOAH <0.5 mg kg^−1^ [28,29]. To render the recycled paper used in this work suitable for food contact, a decrease in the migration for MOSH to at least 80% and for MOAH to at least 5% would be necessary. The LLDPE films would, thus, not show an adequate barrier efficiency for MOSH or MOAH. The PP film showed good barrier efficiency against MOSH, but also not for MOAH. The chitosan acetate film reduced both MOSH and MOAH migration to not detectable. Therefore, the conclusion is drawn that it is an excellent barrier against the migration of MOH.

**Figure 3:**
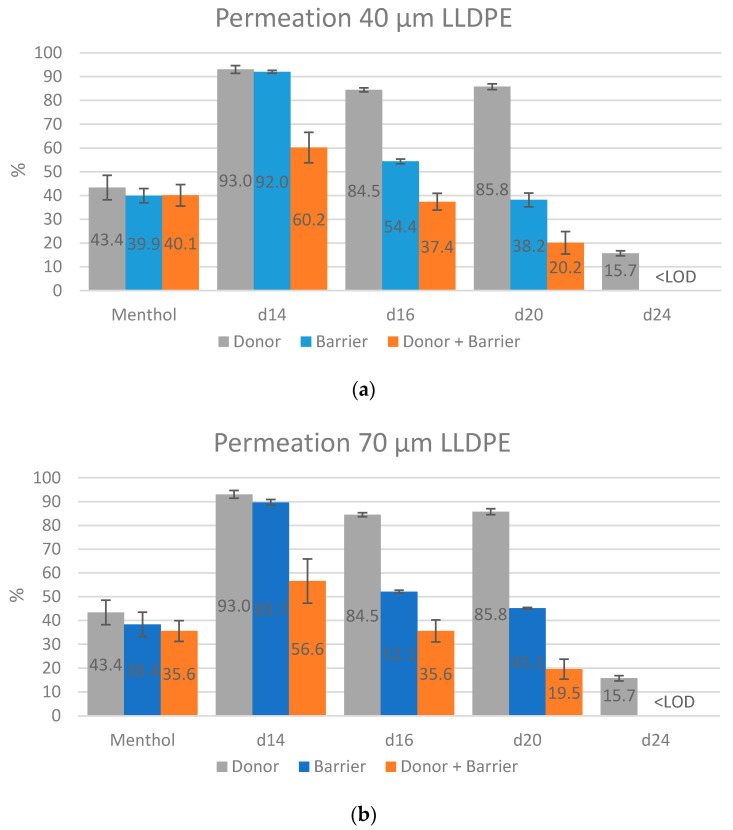

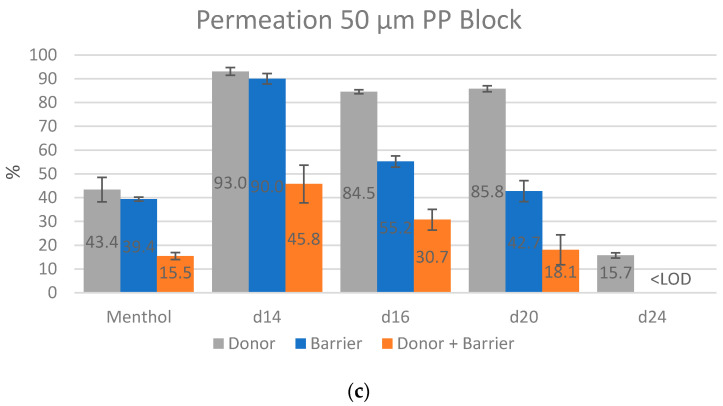
Permeation through samples (**a**) 40 µm LLDPE film, (**b**) 70 µm LLDPE film, and (**c**) 50 µm PP film. *n* = 3.

The last parameter to be evaluated was the permeation through the samples. In theory, testing permeation allows an estimation to be made on the barrier efficiency of materials without applying an additional donor. In practice, the experimental set-up and tests performed both with and without a donor allowed interesting conclusions to be drawn. Figure 3 shows the permeation rates through the donor, the barrier films and the donor plus the barrier films. One trend is clearly visible: By combining donor and barrier the permeation is decreased for all alkanes. However, no clear difference between the polyolefin films can be seen, indicating that those barrier effects are merely the results of the larger sample thickness (=distance to permeate). Furthermore, as already discussed in [19], the polar aroma active substances eugenol, vanillin and acetovanillone, as well as the non-volatile C_28_ did not permeate in amounts above the limit of detection.

In more detail, the barrier films showed no barrier effects whatsoever for menthol and d-C_14_. The combination of donor and film, therefore, also resulted in no effect for menthol, except for the PP film. The permeation of the alkanes decreases with increasing chain length: Only a slight effect can be seen for C_14_, while permeation of d-C_16_ and d-C_20_ is decreased to about 40% and the one of C_24_ is low in general and decrease to <LOD with the films. Combining the donor and the barrier film led to an increase of this volatility effect, since the distance to overcome is greater. The decrease in permeation values of about 80% for, for example, d-C_20_ are; thus, not the result of good barrier efficiency and the three tested films not appropriate for barrier use. The results are in accordance with the studies published earlier [16,17,18].

In contrast, the chitosan acetate film decreased the permeation for all model substances again to not detectable.

## 3. Materials and Methods

### 3.1. Materials, Reagents and Standards

Most of the materials used were purchased from Macherey-Nagel GmbH&Co. KG (Düren, Germany), such as 30 mL glass vials with screw cap equipped with silicone/PTFE septa (N22; 3.2 mm) for extraction of MPPO, 1.5 mL glass vials with screw cap equipped with silicone/PTFE septa (N9; with hole; 1 mm) and micro inserts (15 mm tip; for wide opening; 0.2 mL/6 × 31 mm; clear) for the measurements of extracts. Filter papers used were MN 615 ¼ of 150 mm diameter and were washed with n-hexane and stored at 100 °C overnight in an oven to reduce their MOH contamination, which was derived from their recycled board packaging. Pasteur pipettes were from Fisherbrand, made of glass, unplugged and of 150 mm length from Fisher Scientific GmbH (Schwerte, Germany). Micropipettes of different volumes were used (1 µL–200 µL) for, for example, standards dilution. They were supplied from BLAUBRAND^®^, intraMARK, DE-M, CE, BRAND GMBH + CO KG (Wertheim, Germany). The climate oven used for migration and permeation experiments was an APT.line^®^ KBF-ICH climatic chamber for constant conditions with program control, and with ICH compliant illumination in the doors from Binder GmbH (Tuttlingen, Germany). The automatic solvent evaporator used was a TurboVap^®^ II (Biotage, Uppsala, Sweden) operated at 40 °C water bath temperature and a nitrogen stream of 1–10 bar, depending on the solvent to be evaporated. Evaporation tubes with a total volume of 50 mL and a 0.5 mL end-point from the same supplier were used. MPPO, a poly 2,6-diphenyl-p-phenylene oxide (Tenax^®^ TA (refined), 60–80 mesh; SUPELCO, Bellefonte, PA, USA) was used as a simulant for dry, non-fatty food. It was applied to the samples in an amount of 4 g dm^−2^. All solvents used were purchased in glass bottles: Acetone (ROTISOLV^®^ ≥99.9%, UV/IR-Grade) and dichloromethane (ROTISOLV^®^ ≥99.9% GC Ultra Grade) were purchased from Carl Roth GmbH + Co. KG (Karlsruhe, Germany), n-hexane (Picograde^®^ for Residue Analysis) from LGC Promochem GmbH (Wesel, Germany).

Two internal standard mixes were used, as described in [22]: The first one was used to control the extraction of MPPO, called migration internal standard mix, and consisted of the following substances in a concentration of 200 mg L^−1^ each in acetone: *n*-dodecane-d_26_ (d-C_12_; EURISOTOP SAS, Saint-Aubin, France), *n*-nonadecane-d_40_ (d-C_19_; 98%; Cambridge Isotope Laboratories, Inc.; Tewksbury, MA, USA), benzophenone-d_10_ (d-Bzp; C_13_D_10_O; 99atom%), *bis-*(*2*-ethylhexyl)phthalate-*3,4,5,6*-d_4_ and *di*-*n*-butylphthalate-*3,4,5,6*-d_4_ (d-DEHP and d-nBP; both “analytical standards”) were purchased from Sigma-Aldrich Co. (St. Louis, MO, USA). A total of 25 µL of this stock solution was added before the extraction of the food simulant. The second one was added to the extract prior to the analysis of mineral oil hydrocarbons using online-coupled HPLC-GC-FID. It was purchased by Restek Corporation (Bellefonte, PA, USA) and contained the following substances in 1 mL ampules in toluene: *n*-undecane (C_11_; 300 µg mL^−1^), *n*-tridecane (C_13_; 150 µg mL^−1^), cyclohexylcyclohexane (CyCy; 300 µg mL^−1^), cholestane (*5*-*alpha*-cholestane; Chol; 600 µg mL^−1^), *1*-methylnaphthalene (1-MN; 300 µg mL^−1^), *2*-methylnaphthalene (2-MN; 300 µg mL^−1^), *n*-pentylbenzene (5B; 300 µg mL^−1^), perylene (Per; 600 µg mL^−1^) and 1,3,5-tri-*tert*-butylbenzene (TBB; 300 µg mL^−1^). A total of 10 µL was added to the samples to reach a final concentration of 1.5–6 µg mL^−1^.

The permeation standard mix was used to simulate permeation through the samples in two-sided migration experiments and consisted of following substances in a concentration of 100 mg L^−1^ each in acetone: *n*-tetradecane-d_30_ (d-C_14_; 98%), *n*-hexadecane-d_16_ (d-C_16;_ 98%), *n*-eicosane-d_42_ (d-C_20;_ 98%) and *n*-tetracosane-d_50_ (d-C_24;_ 98%); all purchased at Cambridge Isotope Laboratories, Inc. (Tewksbury, MA, USA), of *n*-octacosane-d_58_ (d-C_28;_ CDN Isotopes), and of l- menthol (99%), eugenol (99%), vanillin (97%) and acetovanillone (≥98%) purchased from Sigma-Aldrich Co. (St. Louis, MO, USA). A total of 100 µL of the standard was spiked in the bottom of the migration cell.

For determination of integration fractions in HPLC-GC-FID, a “C_7_-C_40_ Saturated Alkanes Standard” from Supelco (Bellefonte, PA, USA) was used. It contained each n-alkane between C_7_ and C_40_ in a concentration of 1000 mg L^−1^ in hexane. It was further diluted using n-hexane to a working solution of 10 mg L^−1^ for GC-FID and GC-MS measurements and to 1 mg L^−1^ for HPLC-GC-FID measurements.

### 3.2. Instrumentation

#### 3.2.1. GC-FID

Separation was performed using a Hewlett Packard 6890 Series GC system equipped with an Optima delta-6 capillary column (7.5 m × 100 µm × 0.10 µm, Macherey-Nagel, Germany). The oven was programmed to 60 °C (hold 1 min) and raised at 15 °C min^−1^ to 300 °C (3 min). The carrier gas was hydrogen with a linear velocity of 48 cm s^−1^. Aliquots of one microliter were injected with a split of 1:20; injection port temperature was set to 280 °C. The detector temperature was set to 320 °C, air flow was 450 mL min^−1^, hydrogen flow 40 mL min^−1^ and make up gas was nitrogen with a flow of 25 mL min^−1^. Data evaluation was done using “GC ChemStation” version B.04.03 [16].

#### 3.2.2. GC-MS

A GC with MS detection was used to determine the specific migration of individual substances. Gas chromatographic separation was performed using a Shimadzu GC2010 system equipped with a Rxi-5Sil MS capillary column (30 m × 250 µm × 0.25 µm). Helium was used as carrier gas in linear velocity flow control mode with a constant flow of 40 cm s^−1^. Aliquots of 1 µL were injected using a high-pressure splitless injection with 250 kPa for 0.8 min and a column flow of 4.74 mL min^−1^; a gas saver was switched on in a ratio of 1:5 after 2 min. The septum purge flow was 3 mL min^−1^, the injection temperature 270 °C and sampling time 1 min. The initial oven temperature was 70 °C (1 min) and was raised at 6 °C min^−1^ to 340 °C (5 min). Detection was done with GCMS-QP2010 PLUS mass selective detector. Ions were generated with electron ionization (70 eV), the detector voltage was relative to tune, mass spectrometer scanned from *m/z* 35 to 500 with a scan rate of 1666 amu s^−1^ and an event time of 0.3 s. The software used was “GC-MS Solution” version 4.42.

#### 3.2.3. HPLC-GC-FID

The HPLC was a Shimadzu LC 20AD equipped with an Allure Silica 5 µm column (250 × 2.1 mm). Gradient elution was used, started with 100% *n*-hexane (flow 0.3 mL min^−1^) and raised to 35% dichloromethane within 2 min (hold for 4.20 min). The column was back-flushed at 6.30 min with 100% dichloromethane (flow 0.5 mL min^−1^; hold for 9 min) and reconditioned to 100% *n*-hexane (flow 0.5 mL min^−1^; hold for 10 min). Flow was decreased afterwards to 0.3 mL min^−1^ until next injection. The UV-detector was equipped with a D_2_-lamp set a 230 nm and 40 °C cell temperature.

The GC was a Shimadzu GC 2010 dual-column and dual-FID system, equipped with two guard columns (Restek MXT^®^ Siltek (10 m × 0.53 mm id)) and two analysis columns (Restek MTX^®^-1 (15 m × 0.25 mm id × 0.1 µm df)). Carrier gas was hydrogen with 150 kPa analysis pressure and an evaporation pressure of 87 kPA for MOSH and 85 kPA for MOAH. The oven was programmed to 60 °C (hold 6 min), raised at 20 °C min^−1^ to 100 °C (0 min) and followed by 35 °C min^−1^ to 370 °C (9.29 min). The LC-GC interface was controlled by “Chronect-LC-GC” by Axel-Semrau. Data evaluation was done using “LabSolutions” of Shimdazu Corporation for LC and GC data in version 5.92.

#### 3.2.4. Comprehensive 2D-GC×GC-MS

The comprehensive GC×GC-MS data were collected using the following instruments and settings: An OPTIC-4 Multimode GC Inlet was used programmed in PTV mode at 40 °C (5 s) and raised to 340 °C with 15 °C/s. The autosampler was an AOC- 5000 Plus, the gas chromatograph was a Shimadzu GC-2010 Plus for mass spectrometer. The column combination used was a Rxi^®^-5 MS (30 m × 0.25 mm × 0.25 µm) in the first dimension and a BPX50 (2 m × 0.15 mm × 0.15 µm) in the second dimension. The columns were placed in the same oven. The initial oven temperature was 60 °C (2 min) and was raised to 320 °C (5 min) with a ramp of 5 °C min^−1^. Aliquots of one microliter were injected in splitless mode using a high-pressure injection pulse of 250 kPa for 1 min and a sampling time of 2 min. Helium was used as a carrier gas with 1.8 mL min^−1^ constant flow. The modulator used was a cryogenic modulator (Zoex ZX1 two-stage loop thermal modulator; Zoex Corporation, Houston, TX, USA). Modulation frequency was 7 s with a hot-jet pulse of 700 ms. The temperature of the hot-jet was programmed at 250 °C (25 min), followed by 300 °C from 25–40 min run time and 350 °C from 40 min till the end of the run. The cold jet flow was 10 L min^−1^. Detection was done using a mass spectrometer GCMS-QP2010 Ultra. Ions were generated with electron ionization (70 eV). The detector voltage was 0.85 kV. The ion source temperature was set to 200 °C, the interface temperature at 300 °C. The mass spectrometer was scanned with a scan speed of 50 scans s^−1^ from 54 to 300 m/z. The software used for data evaluation was Version 2.2 SP1 of “ChromSquare”.

### 3.3. Barrier Films Used

For the man-made chitosan acetate film, industrially produced chitosan powder composed of particles with a diameter <200 µm was kindly supplied by a chitosan producer from Germany. According to the product data sheet provided by the producer, the degree of deacetylation of chitosan acetate used is 90% and average molecular weight is 115 kDa. The chitosan acetate was produced from the carapace skin of crustaceans and the ash content is below 1% (*w*/*w*). The dynamic viscosity of a 1% (*w*/*w*) chitosan acetate solution at standard conditions is 135 mPa.s. For the barrier measurements, free standing films were made according to [30,31]. The films had a thickness of 30 µm (±1.5 µm) and were made by dissolving chitosan powder in heated deionized water (60 °C). Chitosan powder was added in small quantities (gram by gram) and stirred for 6 h at 500 rpm. In order to reach full solubility of chitosan in water, acetic acid (100%, Rotipuran, Carl Roth GmbH+Co. KG) was used as an additive in order to acidify water to the pH of 4. After dissolving the chitosan, a clear and yellow tinted chitosan acetate solution with a concertation 2% (*w*/*w*) was obtained without visible particles. In order to obtain clear and homogenous films, any air bubbles must be removed from the chitosan acetate solution and for this purpose the solution was placed into an exicator, whereby vacuum was applied to remove air bubbles. Subsequently the chitosan acetate solution for the free-standing films was poured onto polystyrene Petri dishes and dried at ambient temperature for 48 h. After 48 h chitosan acetate films were climatized in climate chamber at 23 °C and 50% relative humidity.

Three conventionally produced polymer films were compared to the lab-made chitosan acetate film: a 40 µm linear, low density polyethylene film (LLDPE), a 70 µm LLDPE film and a 50 µm polypropylene film (PP) were received from a local producer.

### 3.4. Two-Sided Migration Experiment

A two-sided migration experiment was used to characterize the materials themselves and their barrier properties as described in [22]. In short, migration experiments were performed in triplicates using migration cells (MigraCell^®^ MC 60; contact area ~0.5 dm^2^; FABES Forschungs-GmbH, Munich, Germany). To cut the samples into appropriate circles, scissors and as template, the metal plate of the cell were used. The cell was assembled for two-sided migration experiments, as described in [22]: A piece of cellulose was placed at the bottom part of the cell, on which the modelling substances for the permeation were spiked and the groove of the cell equipped with a FEP-coated O-ring. The samples were placed onto the bottom part and the cell closed with the upper lid, fitted with a red FEP-coated O-ring. The cell was fixed with a clamping ring. The sample was covered with 2 g MPPO through the screw plug on the top of the cell. Acceleration of the migration experiments was carried out by using standard testing conditions of 40 °C for 10 days [8]. Subsequently the MPPO was drained into a glass vial with a screw cap and 25 µL of migration internal standard mix were added. The MPPO was extracted three times with 10 mL of *n*-hexane and 3 min of vortexing. The extracts were combined through a folded filter in a 50 mL evaporation vial and the solvent evaporated to 0.5 mL in an automatic solvent evaporator. The extract was then transferred into 1.5 mL autosampler glass vials with screw cap; 0.5 mL of fresh hexane used to wash the evaporation vial and the solvent combined with the initial extract to achieve an end volume of 1 mL. All extracts were stored in a refrigerator. A small quantity of the extract only was filled into a 1.5 mL glass vial with a screw cap and a micro inlet. The extracts were measured on GC-FID to determine the overall migration of volatile compounds, on GC-MS to determine the permeation of the model substances spiked in the bottom of the cell and on the HPLC-GC-FID to determine the migration of MOSH and MOAH, as described above. GC×GC-MS was used to generate further information on the samples. Detailed information on the experimental set-up, consideration of testing conditions and validation of the method are given in [22].

The four barrier films were characterized using the following test series: To determine the migration of substances initially present in the films and the permeation through the film, a two-sided migration experiment was done without an additional donor first (barrier film + food simulant). Afterwards, a recycled raw paper was inserted as a donor to simulate a real packaging situation (recycled paper + barrier film + food simulant). The recycled paper used as donor was pre-characterized earlier using the same experimental set-up. The MPPO was extracted and the measurements were made as described above.

## 4. Conclusions

The main aim of this study was, on the one hand, to compare the barrier properties of chitosan acetate with the ones of conventionally used polymers and, on the other hand, to further evaluate the capability of the two-sided migration experiment, introduced shortly before.

The two-sided migration experiment allowed to establish a comprehensive characterization of the materials itself and its barrier properties. The possibility for using recycled paper as a donor provides the opportunity to generate data in realistic packaging situations, as it was done in this study for four barrier films.

The conventional polyolefin films showed modest barrier efficiency against the migration of MOSH and MOAH, as already discussed in earlier studies. The effects, as seen, are mainly related to the increased permeation pathway and the volatility limitations. Furthermore, the polyolefins showed a high intrinsic POSH migration. By contrast, the chitosan acetate film showed excellent properties against MOH migration and permeation through the film, which were reduced to not detectable. Furthermore, it showed no intrinsic migration. Together with its excellent film-formation properties, it is singling out as a good bio-based and bio-degradable alternative to conventional materials. Although it is known to be water soluble, it can be used as functional barrier for dry-non-fatty food or in secondary or tertiary cellulose-based packaging materials, since direct contact to moisture should not occur in those applications. In conclusion, the biopolymer is a promising material for future applications.

## Figures and Tables

**Table 1:** Overall migration for barrier films without donor (intrinsic; representing polyolefin oligomeric saturated hydrocarbons (POSH)) and films with donor (POSH + mineral oil saturated and aromatic hydrocarbons (MOSH/MOAH)). Values normalized to the values of the donor (migration of donor = 1). Limit of detection (LOD) = 0.15. *n* = 3.

[1]	40 µm LLDPE	70 µm LLDPE	50 µm PP	Chitosan Acetate
without donor	0.96 ± 0.09	1.08 ± 0.10	0.50 ± 0.02	<LOD
with donor	1.04 ± 0.09	1.17 ± 0.15	0.76 ± 0.06	<LOD

* LLDPE = linear low-density poly ethylene; PP = poly propylene.

**Table 2:** Barrier properties of polymer films against the migration of MOSH and MOAH from a recycled paper donor; given in % of donor. *n* = 3.

Migration [%]	Donor	40 µm LLDPE	70 µm LLDPE	50 µm PP	Chitosan Acetate
MOSH	89.8 ± 10	86.0 ± 4.2	85.4 ± 2.6	58.9 ± 5.5	<LOD
MOAH	10.2 ± 1.9	7.04 ± 0.3	6.8 ± 0.3	6.6 ± 0.7	<LOD
Sum	100 ± 12	93.1 ± 4.5	92.2 ± 2.9	65.4 ± 6.2	<LOD
